# Functionally mediated cranial allometry evidenced in a genus of rock-wallabies

**DOI:** 10.1098/rsbl.2024.0045

**Published:** 2024-03-27

**Authors:** D. Rex Mitchell, Sally Potter, Mark D. B. Eldridge, Meg Martin, Vera Weisbecker

**Affiliations:** ^1^ College of Science and Engineering, Flinders University, GPO Box 2100, Adelaide, South Australia 5001, Australia; ^2^ Australian Research Council Centre of Excellence for Australian Biodiversity and Heritage, Wollongong, New South Wales 2522, Australia; ^3^ School of Natural Sciences, Macquarie University, Sydney, New South Wales 2109, Australia; ^4^ Australian Museum Research Institute, Sydney, New South Wales 2010, Australia

**Keywords:** geometric morphometrics, skull, biomechanics, Macropodidae, evolution

## Abstract

In assessments of skeletal variation, allometry (disproportionate change of shape with size) is often corrected to examine size-independent variation for hypotheses relating to function. However, size-related trade-offs in functional demands may themselves be an underestimated driver of mammalian cranial diversity. Here, we use geometric morphometrics alongside dental measurements to assess craniodental allometry in the rock-wallaby genus *Petrogale* (all 17 species, 370 individuals). We identified functional aspects of evolutionary allometry that can be both extensions of, and correlated negatively with, static or ontogenetic allometric patterns. Regarding constraints, larger species tended to have relatively smaller braincases and more posterior orbits, the former of which might represent a constraint on jaw muscle anatomy. However, they also tended to have more anterior dentition and smaller posterior zygomatic arches, both of which support the hypothesis of relaxed bite force demands and accommodation of different selective pressures that favour facial elongation. By contrast, two dwarf species had stouter crania with divergent dental adaptations that together suggest increased relative bite force capacity. This likely allows them to feed on forage that is mechanically similar to that consumed by larger relatives. Our results highlight a need for nuanced considerations of allometric patterns in future research of mammalian cranial diversity.

## 1. Introduction

Allometry in morphology refers to disproportionate changes in shape that correlate with changes in size [1–3]. These changes can be ontogenetic, relating to genetically determined developmental processes; static, associated with individual differences in growth and development influenced by genetic and environmental variations; or evolutionary, which often extend from static allometry, but can also be influenced by size-correlated functional adaptations across species [4–7]. Despite this latter functional aspect, allometry has rarely been viewed from a functional perspective in analysis and interpretations. Instead, it is often treated as an expected pattern produced through vaguely defined developmental constraints. The effects of allometry are usually then simply accounted for in comparative analyses with little further discussion, and removed under the assumption that functional traits should be evident independent of size. However, with regards to the mammalian cranium (the skull without the mandible), many allometric patterns are in fact related to size-mediated trade-offs in functional demands [8].

A larger species can bite harder than a smaller species because larger-sized individuals generally also have bigger skulls, jaw muscles and teeth [8–12]. If two mammalian species share a common cranial structure (i.e. a common ecomorphology [8,13,14] within a phylogenetically defined ‘ordinal Bauplan’ [15]), and similar feeding or biting behaviours, the larger species is therefore expected to exhibit more elongate (or ‘gracile’) craniofacial proportions. Such craniofacial morphology reflects lower bite force demands, meaning its evolution has instead responded to other selective pressures that favour a more elongate facial skeleton. Conversely, the cranium of the smaller species is likely to reflect a functional emphasis on bite force capacity in their cranial morphology [8]. This ‘bite force allometry’ is especially clear across extant mammalian herbivores, evidenced by the longer, more gracile skulls of larger species [8,13,16–19], and is likely to be a major influence on mammalian cranial diversity in general [8]. However, ontogenetic, static and evolutionary allometry are often correlated and not easily distinguished from each other [8], potentially leading to some functional aspects of morphology being missed through the dismissal of allometry as a determinant of function in morphological studies. Therefore, more detailed interpretations of allometry are needed to differentiate the functional aspects of size variation from other patterns.

**Figure 1.**
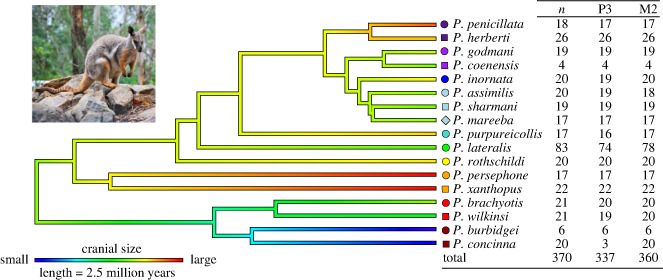
*Petrogale* is a genus of rock-wallabies comprising 17 species distributed across Australia. The sample of 370 *Petrogale* used in this study is presented on the right. *n* = Sample for shape analysis, P3 = sample for premolar analysis, M2 = sample for molar analysis. Symbols and their coloration delineate monophyletic clades and sister taxa for phylogenetic reference in figures 2 and 3.

*Petrogale* (a genus of rock-wallabies) is an ideal group to identify functional allometric patterns in the mammalian cranium. The genus is a product of recent and rapid diversification [20]. It comprises 17 species ranging from approximately 1.5 kg to approximately 12 kg [21–24], including a monophyletic clade of two dwarf species (figure 1). The species that have had their diets assessed are considered ‘mixed feeders’, incorporating a variety of vegetation types such as grasses, shrubs, and herbs into their diets with no obvious specialization [25,26]. An order of magnitude difference in body size variation, alongside generally similar, unspecialized feeding ecology, provide excellent conditions for identifying both relaxed bite force demands in larger species, and adaptations for increased bite force in the smallest species.

We base our hypotheses on the predictions of Mitchell *et al*. [8], in that the crania of larger *Petrogale* species should exhibit at least some of the following: narrower bizygomatic breadths, representing lower relative muscle force; a lower ratio of in-lever to out-lever, reflected in either retracted musculature (in-lever) or a projected dentition (out-lever); and less robust (i.e. more gracile) regions of the facial skeleton for dispersing lower bone stress experienced during biting. By contrast, the smallest species should show a reverse pattern with a more stout overall shape of the cranium, thereby increasing capacity for harder biting relative to their size. Given that relatively larger teeth are often seen in mammals that feed on more resistant foods [27–29], we also expected the dwarf species to have relatively larger teeth that accommodate a broader diet for their size.

## 2. Material and methods

To investigate cranial evolutionary allometry across *Petrogale*, we elaborated on the methods defined by [8], involving a geometric morphometrics approach on three-dimensional coordinate data.

We surface-scanned 370 *Petrogale* crania using a Polyga Compact S1 scanner or HDI109 blue light scanner. All were from adults, determined by M4 molar eruption [16] (see electronic supplementary material, table S1 for specimen details). We processed scans in Geomagic Wrap (v. 2021) and allocated landmarks using Checkpoint (Stratovan). The landmarking protocol used a combination of fixed and semi-sliding landmarks (electronic supplementary material, table S2). Notably, the molars of kangaroos and wallabies (typically limited to four in each tooth row) erupt from the rear of the maxillae and migrate forward throughout their development and adult life [30–32]. This characteristic migration of the cheek tooth row is known as ‘molar progression’. Because of molar progression, fixed landmarks representing specific cheek teeth would introduce unnecessary variation. Instead, the cheek tooth row was defined by the anterior limit of the premolar (or margin of premolar loss), and the point where the molars erupt from the maxillae.

We imported landmark data into R Studio (v. 2023.03.0) and analysed it with R (v. 4.2.1) [33] using the ‘geomorph’ package [34]. A Procrustes superimposition was performed on the raw coordinates [35]. We additionally removed variation due to asymmetry.

To first identify allometric variation across the genus, we did a Procrustes multivariate analysis of variance (MANOVA) using the Procrustes tangent coordinates as response variables and the natural logarithm of cranial size as the predictor variable. Size was represented by the centroid size [36]. We visually assessed predicted shape changes associated with size using landmark vector displacements [37,38] with the ‘landvR’ package [39] and assessed this relationship across the genus by plotting cranial shape against log(size). To investigate in more detail how evolutionary allometry relates to static/ontogenetic allometry, we tested a model with species as covariate and plotted this relationship with regression lines for each species. We then tested interspecific differences in static allometry by comparing the slopes and angles of allometric regressions in multivariate space between species [40,41] and explored allometry via size-related shape predictions for each species.

We then performed principal component analysis (PCA) on the Procrustes shape data. The first five components were individually regressed against cranial size to determine if they represented orthogonal allometric trajectories that together contribute to the predicted shape changes.

We used a single individual from each species and 1961 nuclear loci from [42] to generate a time-calibrated phylogenetic tree using MCMCTree [43]. The phylogeny was estimated using MCMCTree [43] using the concatenated nuclear dataset. We estimated the phylogeny and divergence times using soft constraints which we used from previous fossil calibrated phylogenetic results from [20]. We included a root age 6–11.3 million years ago and included four secondary calibrations: one for the *brachyotis* group node (1.9–5.6 mya); one for the *penicillata* group excluding *P. herberti* and *P. penicillata* (900,000–2.7 mya); one for the clade including *P. rothschildi, P. lateralis, P. purpureicollis* and the *penicillata* group (4.4–5.5 mya); and one including all species except those in the *brachyotis* group (1.9–8.7 mya). The analysis was run using the HKY nucleotide substitution model, 0.5 alpha for gamma rates at sites and four discrete gamma categories. We used an independent rates model, the approximate likelihood calculation [44] and kept missing sites in the analysis. We allowed 1 million generations as burnin, then sampled every 1000 generations for 10 000 samples. We assessed convergence using Tracer v. 1.7.2 [45] and effective sample sizes greater than 200. In order to test for a phylogenetic influence on allometry, we then did a phylogenetic generalized least squares (PGLS) analysis with the mean shape of each species as the response variable, the natural logarithm of the mean cranial size for each species as the predictor, and a time-calibrated tree of the genus (figure 1). We then tested for a phylogenetic signal of cranial size to determine if size was phylogenetically constrained. We visualized distributions of cranial size across the phylogeny with the ‘phytools’ package [46] (figure 1).

To compare relative tooth sizes, we measured anteroposterior length of the P3 premolars and M2 molars (when present; figure 1). We then divided the lengths by cranial size, allowing examination of the relative size of the teeth for each species. We performed ANOVAs with these dental ratios as the response variable and species as the predictor. Species differences were plotted using the ‘ggplot2’ package [47].

## 3. Results

**Figure 2.**
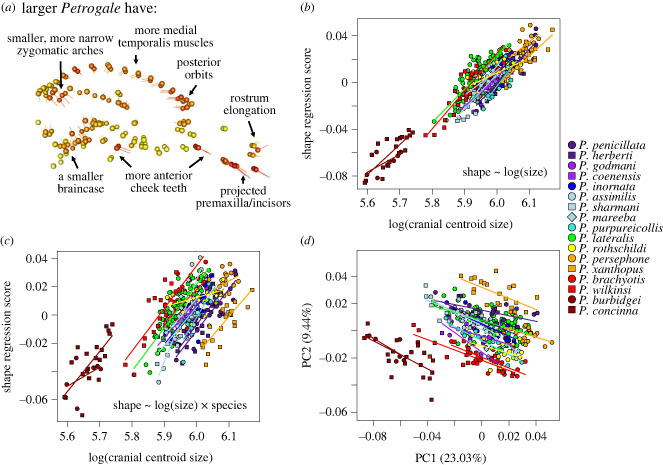
(*a*) Allometric shape predictions across the entire sample. Orbs represent the predicted shape for the smallest crania, while the tips of the vector lines represent the predicted shape of the largest crania. Labels indicate shape changes that occur with increased size. (*b*) Regression of multivariate shape and size. Species regression lines are included for clarity, but are not represented in the model. (*c*) The full model including species and regression lines. (*d*) PCA of shape variation. Regression lines indicate intraspecific negative correlations between PC1 and PC2.

The OLS regression revealed significant allometry across the sample (*R*^2^ = 0.173, *F*_1,368_ = 76.851, *p* = 0.001). Shape predictions indicate that larger crania across the genus should have smaller braincases with more posterior orbits, a projected rostrum mostly involving the incisor arcade, a functional cheek tooth row positioned more anteriorly, and a narrower bizygomatic breadth with smaller, more anteriorly positioned superior zygomatic roots of the squamosals (figure 2*a*).

All species had significant intraspecific allometry, except the two species with very low sample sizes (*P. burbidgei* and *P. coenensis*; figure 1) and *P. persephone*, possibly due to a narrower range of sizes among the larger individuals of the entire sample (see electronic supplementary material, figure S1 for all static allometry test results and shape predictions). The similar allometric trajectories of all species can be seen in the plots of generic allometry (figure 2*b*) and the full model that included species as a factor (figure 2*c*). The full model had a significant interaction effect between cranial size and species (*R*^2^ = 0.031, *F*_16,336_ = 1.296, *p* = 0.001), indicating that some species had divergent slopes in the relationship between size and shape. Pairwise comparisons revealed these divergent slopes mostly involved *P. assimilis*, *P. concinna* and *P. rothschildi* (figure 2*c*, electronic supplementary material, table S3). Furthermore, analysis of allometric angles (electronic supplementary material, table S4) indicated that, despite the significant interaction effect, species rarely differed statistically in their allometric trajectories. The exceptions were the two species with low sample sizes, but also *P. rothschildi* which exhibited different allometric predictions involving the foramen magnum and rear maxillary region. For the remaining 13 species (also excepting *P. persephone*), static allometry was generally similar across species, with allometric variation dominated by a medial extension of the temporalis muscle attachments, anterior migration of the cheek tooth rows, projection of the rostrum/premaxillae/incisor arcade, narrower bizygomatic breadths, and smaller braincases. However, one noteworthy region of exception was the rear zygomatic root, which showed an anterior shift with increasing size across species, but often a posterior/medial shift within species (electronic supplementary material, figure S1).

The first principal component (PC1; 23.03%) is strongly correlated with cranial size (*R*^2^ = 0.678, *F*_1,368_ = 778.900, *p* < 0.001). PC1 indicates that larger individuals across the sample tend to have smaller braincases with more medial temporal scars, orbits positioned more posteriorly, a smaller rear zygomatic root, projected premaxillae/incisors, and a cheek tooth row positioned more anteriorly (figure 2*d*).

PC2 (9.44%) also correlates with size (*R*^2^ = 0.079, *F*_1,368_ = 32.810, *p* < 0.001) and almost exclusively indicates that larger individuals across the sample tend to have a more anteriorly displaced superior zygomatic root on the squamosal and medially expanded rear temporalis muscle origins. Thus, both PC1 and PC2 together represent the predicted allometric patterns of figure 2*a*. However, there is a clear species-level negative correlation visible between PC1 and PC2 for most species (figure 2*d*), with a *post-hoc* linear regression analysis revealing a significant relationship between PC1 and PC2 when adjusting for species (*R*^2^ = 0.145, *F*_17,352_ = 263.872, *p* = 0.001). The distribution of different species on PC1 and PC2, alongside the *post-hoc* results, indicate that PC2 represents the ontogenetic/static condition of the rear zygomatic root, whereby larger individuals within species often have a more posterior-medial positioning of the superior zygomatic root on the squamosal (electronic supplementary material, figure S1). The contrasting evolutionary allometry of this region is pooled together with other size-related shape changes in PC1.

PC3 (8.21%) (not shown) also correlates with size (R^2^ = 0.073, *F*_1,368_ = 29.98, *p* < 0.001) and appears to represent aspects of the auditory bullae and frontal region, being largely driven by the two dwarf species and *P. lateralis* distributed towards the minimum. Since this trend is not present in the allometric predictions explained little variation in PC3 (7.3% of a PC explaining 8.2% of the overall variation), we do not discuss it further here. No subsequent PCs were correlated with cranial size.

PGLS analysis revealed no significant evolutionary allometry (*R*^2^ = 0.081, *F*_1,15_ = 1.318, *p* = 0.276). But there was a significant phylogenetic signal for cranial size (*K* = 1.252, *p* = 0.002).

**Figure 3.**
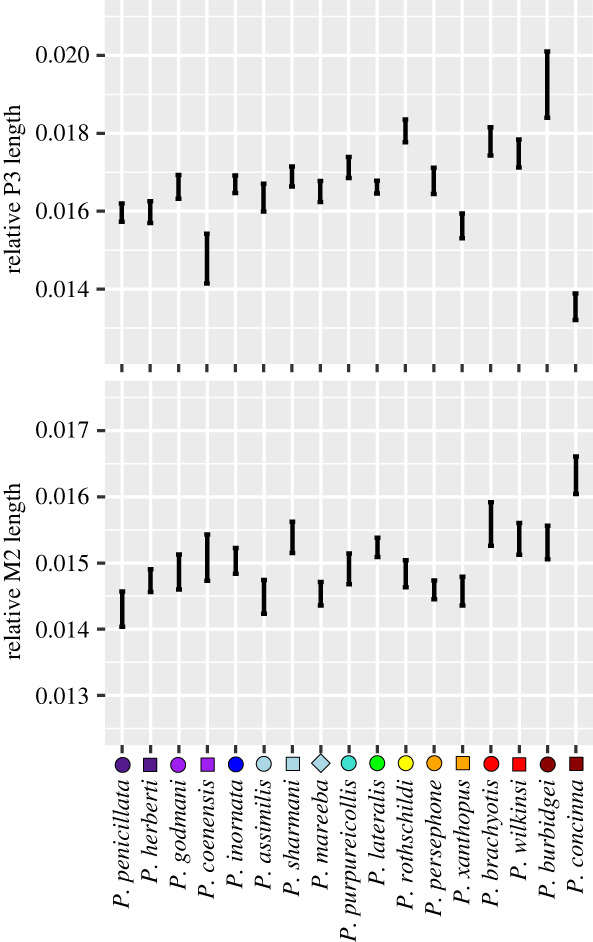
Relative premolar (P3) and molar (M2) sizes in *Petrogale* rock-wallabies.

Figure 3 shows significant differences between species for both relative premolar lengths (*F*_16,320_ = 7.137, *p* = 0.001) and relative molar lengths (*F*_16,343_ = 4.1396, *p* = 0.001). One dwarf species, *P. burbidgei*, had the largest premolars for its size across the genus. By contrast, the other dwarf species, *P. concinna*, had the largest molars and smallest premolars.

## 4. Discussion

Across *Petrogale*, we show that evolutionary allometry can correlate with static allometry positively or negatively, and that these patterns can be portioned into constraints on cranial shape and evidence of functional allometry. Supporting Haller's rule of brain hypoallometry [48], we first identified a relatively smaller braincase and more posterior orbits in larger species, which are allometric patterns common among mammalian taxa and can potentially act as a constraint on the evolution of surrounding craniofacial shape [8,49,50] and increase the extent of temporalis muscle attachment [51]. Aside from this developmental pattern, cranial shape variation within the genus appears to be dominated by changes to biomechanical attributes, whose relations to biting ability are well-known among mammals. We found projected premaxillae in larger species, as is typical in extant mammalian herbivores [8,13,17–19,52] and indicative of poorer leverage when biting with the anterior dentition [16,53,54]. This also occurred alongside more anterior positioning of the entire functional cheek tooth row and a relatively gracile rear zygomatic arch, reflecting our hypothesized attributes for larger species. In an opposing fashion, the two smallest species had larger teeth, alongside a stouter facial skeleton with robust rear zygomatic arches, more capable of producing and accommodating higher relative bite forces. Our hypothesis of allometric adaptation to optimize bite force in smaller species was therefore supported.

We posit that with broadly similar, unspecialized herbivorous diets across the genus, larger species of *Petrogale* can sacrifice some bite force to accommodate functional demands unrelated to bite force capacity. For example, projection of the premaxillae most likely represents a trade-off, whereby sacrificing some bite force capacity can potentially improve food selectivity [55–57] or cropping ability [58,59]. Similarly, the anterior positioning of the cheek teeth in larger species would not sacrifice absolute bite force during slicing with the premolars or mastication with the molars. Rather it can be advantageous because it confers a more balanced bite, thereby decreasing the risk of injury to the jaw joints [29,60,61]. This anterior extension of the tooth row was represented by the anterior limits of the premolar, which acts as a buttress limiting further molar progression [31]. While there was clearly some anterior migration of the tooth rows evident in developmental (ontogenetic/static) allometry within most species, the anterior limits of premolar positioning, and therefore also the migrating molars behind them, were greatest in larger species. Posteriorly, the pattern was evidenced by the location of molar eruption, which was morphologically independent of tooth positioning, therefore indicating a distinct macroevolutionary extension of the molar progression seen in ontogenetic/static allometry.

Evolutionary allometry was inversely correlated with ontogenetic or static allometries in the confined region of the superior zygomatic root. A smaller superior zygomatic root was found in larger species, but larger individuals within species had this region positioned more posterior-medially. This suggests three processes: (1) larger species generally have a less reinforced rear zygomatic root, demonstrating the macroevolutionary moderation of metabolically expensive bone deposition in response to relatively lower reaction forces during biting [8], (2) this also likely results in a relative decrease in the efficiency of vertical muscle force production via a more horizontal orientation of the rear temporalis muscle [62]; and (3) larger individuals within most species have more posterior-medial extension of this feature, probably for better attachment to the relatively smaller braincase.

Both dwarf species had stouter cranial proportions, which confer higher bite forces for size [8,16,29,53,63]. Both also had a functional tooth row positioned more posteriorly, a feature which increases bite force at the cheek teeth [29,60,61,64], and also relatively larger teeth, potentially providing access to a broader range of foods than their size could accommodate under isometric dental scaling. However, we found that both dwarf species each exhibited unique dental adaptations that both support our hypothesis of relatively larger dentition. *Petrogale concinna* has evolved advanced molar progression [31,32]; similar to larger kangaroos, the premolar in *P. concinna* was much smaller and also often lost in adults. This allows the molars to migrate to the front of the toothrow and resulted in low numbers of *P. concinna* with premolars (table 1). Alongside the relatively largest molars of the genus, *P. concinna* also has the only case of supernumerary molars found in marsupials with a seemingly unlimited number of producible molars for continual replacement [21,32,65], where other macropodids are limited to four molars [31]. This means we cannot be certain that the molars measured were M2 molars in all individuals, but the M2's measured in individuals with premolars were no different in relative size to the other measurements of the species. However, *P. burbidgei* had the largest premolar for its size in the genus [66], but no additional molars, thus representing an entirely different approach to increased tooth size, made especially clear by opposing premolar sizes.

Despite being considered generalist mixed feeding herbivores across their present-day distributions, our results suggest *Petrogale concinna* has more specialized dentition for graze and low-level forage with large (and additional) molars to accommodate high dental wear [31,67] and *P. burbidgei* is more specialized for browse with large sectorial premolars for slicing through leaves and twigs [30,31,68]. There is some evidence that this might be related to niche partitioning in *P. concinna*, which has been reported to increase intake of tough spinifex grasses (*Triodia* spp.) during low-productivity seasons, when compared to sympatric *P. brachyotis*, while feeding on a more generalist diet throughout the rest of the year [32] (but also see [23]). However, little information currently exists on dietary ecology of *P. burbidgei* to confirm the occurrence of niche partitioning in this species.

We also found no evidence of allometry when factoring phylogenetic relatedness into the model, as seems to be common when there is a significant phylogenetic signal of cranial size [8]. This result was also detailed by [69] who interpreted this to mean that phylogenetic relatedness explained all variation that would have been attributable to evolutionary allometry without phylogenetic adjustment. However, when relatedness and size are correlated, shape variation attributable to relatedness and allometry are statistically indistinguishable [8], rendering both hypotheses equally feasible. In cases where obvious, ubiquitous allometric effects such as braincase hypoallometry are present alongside a significant phylogenetic signal of size, excluding phylogenetic relatedness from the model appears to be more appropriate for assessing allometric variation [8,70]. But we reiterate that phylogenetic testing is an important diagnostic part of this process and should not automatically be exempt.

In summary, we identified clear morphological evidence of bite force allometry which explains much of the cranial shape variation across the *Petrogale* genus. Patterns of evolutionary allometry can be either an extension of, or inversely correlated with, ontogenetic and static allometry, and are frequently related to functional demands. We argue that more detailed interpretations of allometry should therefore be considered for assessments of mammalian diversity in the future, in order to capture functional aspects of size variation.

## Data Availability

All data collected and analysed are on Github available from the Zenodo Repository: https://zenodo.org/doi/10.5281/zenodo.10525500 [71]. Scans for all crania are available on Morphosource: https://www.morphosource.org/projects/000535920?locale=en [72]. Supplementary material is available online [73].
